# Inhibiting P2Y12 receptor relieves LPS‐induced inflammation and endothelial dysfunction

**DOI:** 10.1002/iid3.697

**Published:** 2022-09-07

**Authors:** Xiuxia Han

**Affiliations:** ^1^ Medical Department of Shandong University Hospital Jinan Shandong China

**Keywords:** clopidogrel, inflammation, migration, P2Y12 receptor, ticagrelor

## Abstract

**Background:**

Acute lung injury (ALI) is characterized by abnormal inflammatory response without effective therapies. P2Y12 receptor (P2Y12R) plays a vital role in inflammatory response. This study intends to explore whether P2Y12R antagonists can inhibit LPS‐induced inflammatory injury of human pulmonary microvascular endothelial cells (HPMVECs) and endothelial cell dysfunction.

**Methods:**

Using a cell model of ALI, the role of P2Y12R was investigated in LPS‐induced HPMVECs. The expression of P2Y12R was detected by RT‐qPCR and Western blot analysis assay and TNF‐α, IL‐1β, and IL‐6 levels were analyzed by RT‐qPCR. NO levels were also analyzed through NO kit. The levels of NF‐κB p65, P‐IκB‐α, and IκB‐α, as well as p‐AKT and eNOS levels were detected by Western blot analysis assay. Wound healing assay was performed to evaluate HPMVECs migration. FITC‐dextran was used to evaluate endothelial cell permeability, and the analysis of adherens junction protein VE‐cadherin and endothelial cell tight junction proteins ZO‐1, Claudin 5 and Occludin expression was performed by RT‐qPCR and Western blot analysis assay.

**Results:**

In vitro, LPS increased the expression levels of P2Y12R and pro‐inflammatory mediators (TNF‐α, IL‐1β, and IL‐6), followed by a decrease in HPMVECs migration. In addition, LPS led to an increase in endothelial cell permeability. P2Y12R antagonists Ticagrelor or clopidogrel treatment significantly reversed these effects of LPS.

**Conclusion:**

The inhibitor of P2Y12R was able to decrease inflammatory response, promote migration and improve endothelial cell function and permeability, suggesting a key role of P2Y12R in ALI.

## Graphical abstract

The inhibitor of P2Y12 receptor was able to decrease inflammatory response, promote migration, and improve endothelial cell function and permeability, suggesting a key role of P2Y12 receptor in acute lung injury.

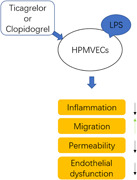

## 1 INTRODUCTION

Acute lung injury (ALI), a condition of acute inflammation, is caused by the destruction of the lung endothelium and excessive neutrophil migration.^1^ Under physiological conditions, pulmonary vascular epithelial cells can inhibit inflammation and blood coagulation. When endothelial cells are exposed to hypoxia, cytokines such as (TNF‐α and IL‐1β), thrombin, and bacterial endotoxins (e.g., LPS) may stimulate or interact with activated inflammatory cells, as a result of which, normal endothelial cells convert to a pro‐inflammatory phenotype.^2^ Lung endothelial cells are important components in the inflammatory response of ALI.^3^, ^4^, ^5^ Elevated lung tissue permeability leads to the formation of pulmonary edema, which is another important pathological feature of ALI.

Platelets can promote the recruitment of neutrophils to the lung leading to lung injury during inflammation.^6^, ^7^ P2Y12 receptor (P2Y12R) is involved in mediating platelet aggregation induced by ADP.^8^ Mutations in the P2Y12R gene have been reported to be related to lung inflammation and asthma.^9^ In addition, P2Y12R deficiency and platelet depletion can eliminate dust‐induced airway inflammation, suggesting that P2Y12 plays a vital role in lung inflammation.^10^ A study has showed that P2Y12R antagonist can inhibit sepsis‐induced lung injury.^11^ P2Y12R antagonists, ticagrelor, and clopidogrel have been found to inhibit NF‐κB signaling pathway to alleviate LPS‐induced venous endothelial dysfunction.^12^

Therefore, this study intends to explore whether P2Y12R antagonists can inhibit LPS‐induced inflammatory injury of pulmonary microvascular endothelial cells (PMVECs) and endothelial cell dysfunction.

## 2 METHODS

### 2.1 Cell culture and treatment

Human PMVECs (HPMVECs) were purchased from the Cell Bank of the Chinese Academy of Science and cultured in DMEM medium (cat. no. SH30081.LS; Hyclone) containing 10% fetal bovine serum (FBS; cat. no. SH30070.01; Hyclone), 100 U/ml penicillin (cat. no. V900929; Sigma‐Aldrich), and 100 μg/ml streptomycin (cat. no. 85886; Sigma‐Aldrich) in a cell incubator with 5% CO_2_ at 37°C. Ticagrelor (cat. no. 274693‐27‐5) and clopidogrel (cat. no. 120202‐66‐6) were both obtained from Sigma‐Aldrich. Cells were incubated with ticagrelor (20 μM) and streptomycin (20 μM) for 12 h.^12^ Cells were stimulated with 100 ng/ml LPS (Invitrogen) for 24 h. Cells were treated with complete growth medium added with 100 ng/ml LPS (cat. no. 00‐4976‐93; Invitrogen),^13^ ticagrelor plus LPS or clopidogrel plus LPS, separately, for 24 h.

### 2.2 Western blot analysis

After adjusting the total protein concentration of the cell samples, 50 μg of protein sample was uploaded per well and separated by polyacrylamide gel electrophoresis. The proteins were transferred to PVDF membrane (cat. no. FFP24; Beyotime) and then washed with PBS (cat. no. C0221A; Beyotime) for three times. After 10 min each, the membrane was blocked with 5% skim milk (cat. no. P0216; Beyotime) at room temperature for 2 h. Next, the membrane was probed with the corresponding primary antibodies at 4°C for incubation overnight and then incubated with secondary antibody Goat Anti‐Rabbit IgG H&L (HRP; ab6721; dilution, 1:2000; Abcam) at 37°C for 2 h. After washing with TBST again, it was placed into Odyssey (LI‐COR) for scanning and analysis. The primary antibodies included P2Y12R (ab233760; dilution, 1:1000; Abcam), NF‐κB p65 (ab32536; dilution, 1:1000; Abcam), P‐IKB‐α (ab133462; dilution, 1:10,000; Abcam), IKB‐α (ab32518; dilution, 1:1000; Abcam), p‐AKT (ab38449; dilution, 1:1000; Abcam), eNOS (ab300071; dilution, 1:1000; Abcam), VE‐cadherin (#2500; dilution, 1:1000; Cell Signaling Technology), ZO‐1 (ab216880; dilution, 1:1000; Abcam), Claudin 5 (ab131259; dilution, 1:1000; Abcam), Occludin (ab216327; dilution, 1:1000; Abcam), H3 (ab1791; dilution, 1:1000; Abcam), and GAPDH (ab8245; dilution, 1:1000; Abcam).

### 2.3 Wound healing

HPMVECs were cultured in DMEM medium with 2% FBS overnight. Next, a scratch was made in cell monolayers with a 200‐µl pipette tip. Serum‐free DMEM was added to wash cells for three times, followed by the addition medium with 2% FBS and treatment with LPS together with ticagrelor or clopidogrel. The images were obtained under an inverted optical microscope.

### 2.4 NO kit

Culture medium was collected for the detection of NO in accordance with the manufacturer's Protocol of NO kit (cat. no. S0021S; Beyotime). Griess reagent was added to each well (50 μl). The absorbance at 540 nm was detected using a microplate reader.

### 2.5 Detection of endothelial cell permeability

HPMVECs were grown into 0.4‐μm transwell inserts. After treatment, FITC‐dextran (1 mg/ml; cat. no. HY‐128868; MedChemExpress) was added to the upper wells. After 30 min, 50 μl of medium in bottom medium was removed. A microplate reader was used to detect the absorbance at the excitation wavelength of 488 nm and the emission wavelength of 520 nm.

### 2.6 Statistical analysis

GraphPad Prism 8.0 statistical software was used for data analysis. The data were expressed as mean ± standard deviation (SD). One‐way ANOVA was used for comparison among groups, followed by Turkey's test.

## 3 RESULTS

### 3.1 P2Y12R antagonists suppress the inflammatory response of LPS‐treated HPMVECs

**Figure 1:**
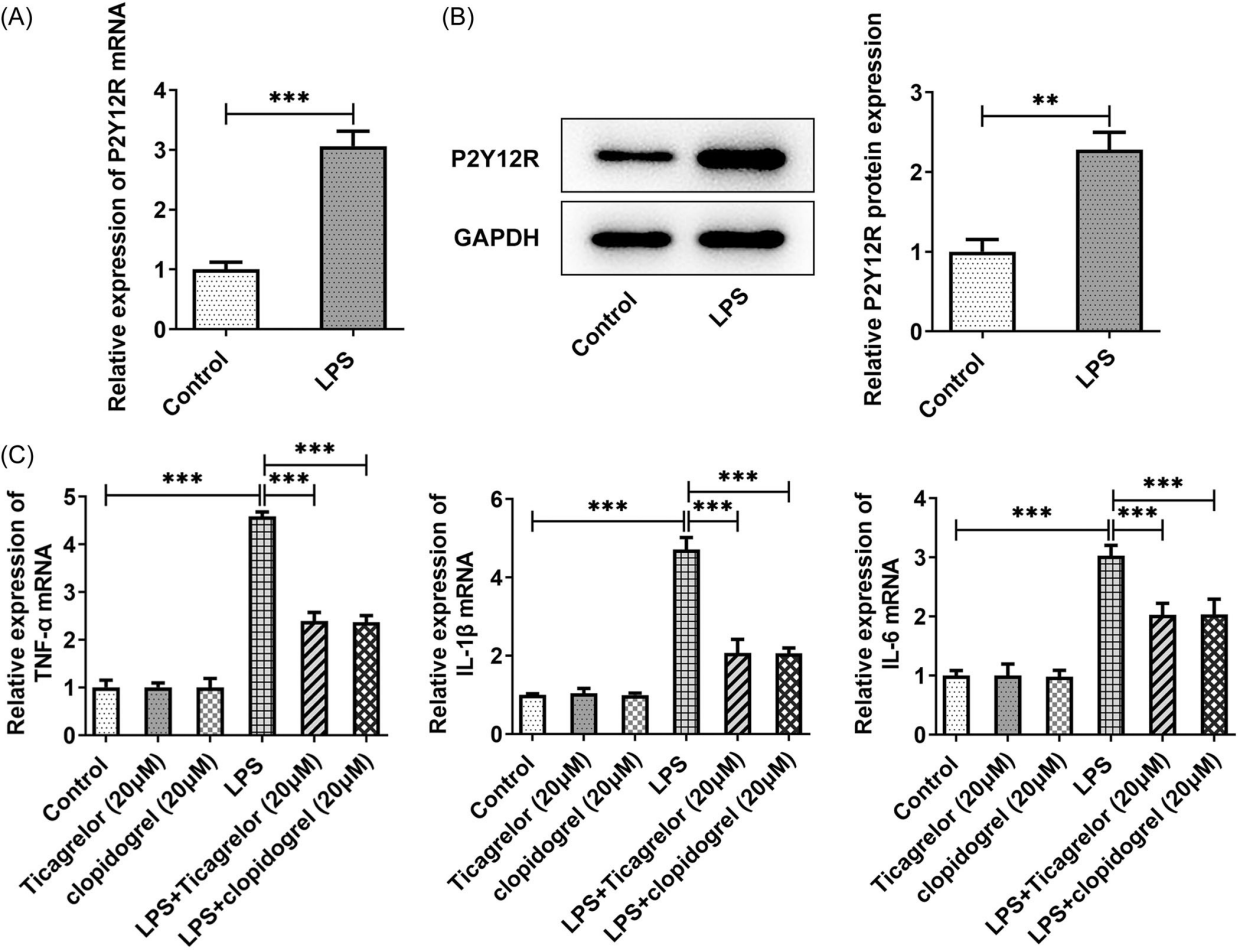
Ticagrelor or clopidogrel treatment decreases inflammatory response induced by LPS in HPMVECs. (A, B) The analysis of P2Y12R levels by RT‐qPCR and Western blot analysis. (C) RT‐qPCR was performed to explore the levels of TNF‐a, IL‐1β, and IL‐6. HPMVEC, human pulmonary microvascular endothelial cell. ***p < .001.

**Figure 2:**
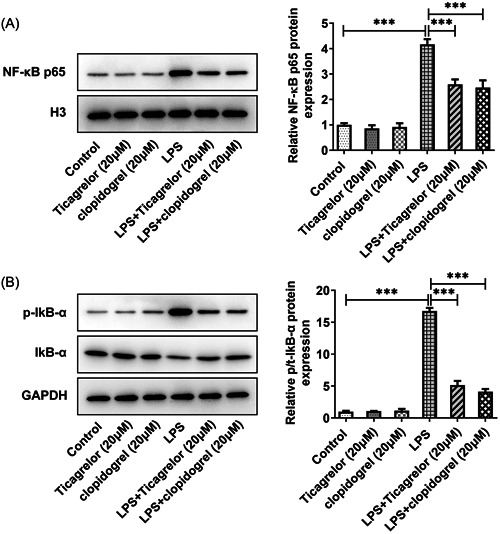
Ticagrelor or clopidogrel treatment modulates NF‐κB/IκB‐α signaling pathway. (A, B) The analysis of NF‐κB p65, P‐IκB‐α, and IκB‐α expression by Western blot analysis assay. ***p < .001.

To study the effect of P2Y12R, HPMVECs were treated with LPS for 24 h. The results showed that LPS treatment increased P2Y12R levels compared with the control group (Figure 1A,B). Ticagrelor or clopidogrel treatment had no significant impact on the levels of TNF‐a, IL‐1β, and IL‐6 in HPMVECs. The levels of TNF‐a, IL‐1β, and IL‐6 were increased after LPS stimulation. But ticagrelor or clopidogrel treatment markedly reduced the levels of these inflammatory factors in LPS‐treated HPMVECs compared with the LPS group (Figure 1C). No significant changes were noted in NF‐κB p65 and p‐IκB‐α levels after ticagrelor or clopidogrel treatment, but a significant decrease was observed in LPS‐treated cells after ticagrelor or clopidogrel treatment (Figure 2).

### 3.2 P2Y12R antagonists promote the suppressed migration and ameliorate dysfunction of HPMVECs upon exposure to LPS

**Figure 3:**
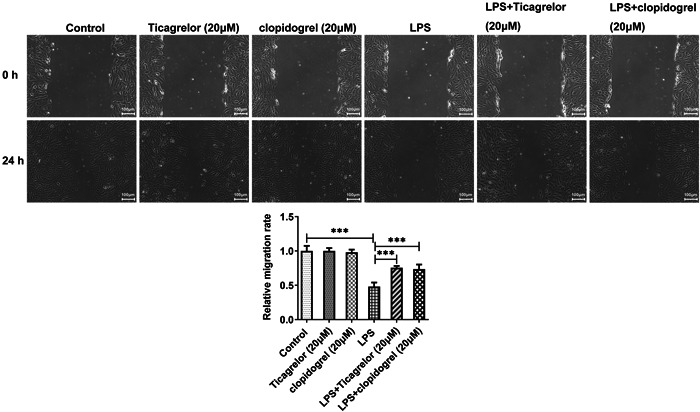
Ticagrelor or clopidogrel treatment increases HPMVECs migration following LPS challenge. Wound healing assay evaluated HPMVECs migration. HPMVEC, human pulmonary microvascular endothelial cell. ***p < .001.

**Figure 4:**
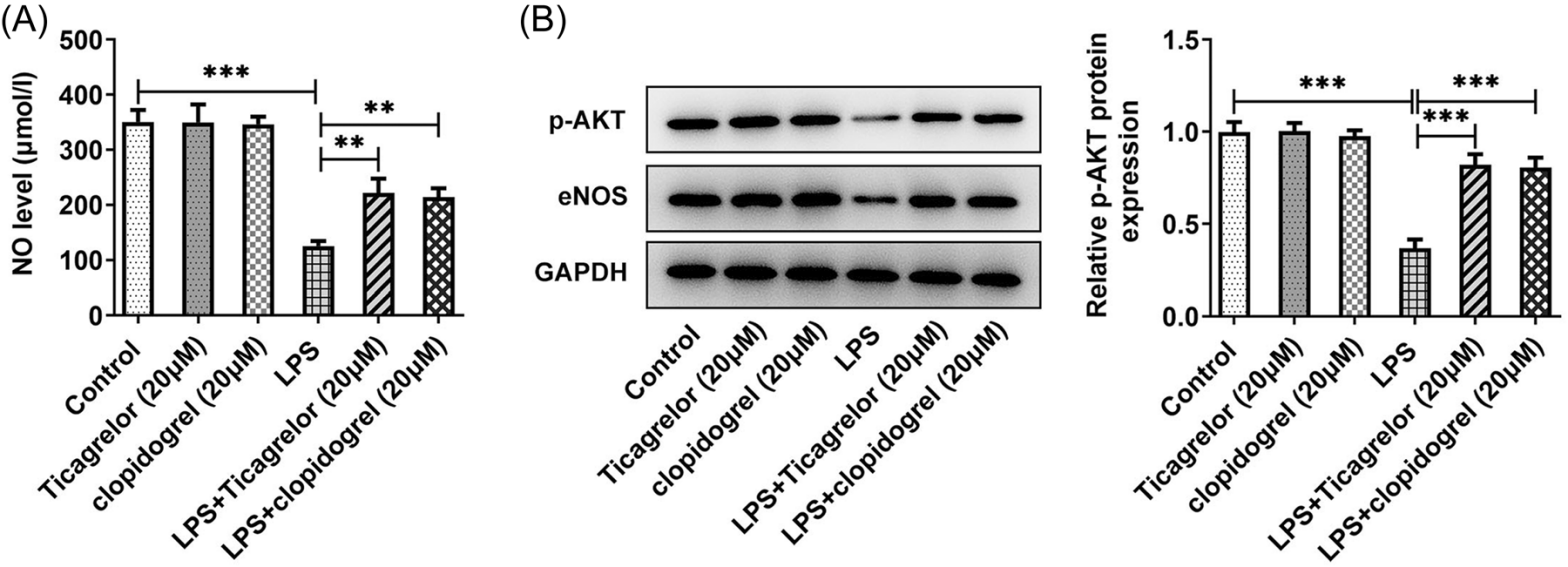
Ticagrelor or clopidogrel treatment improves dysfunction of HPMVECs following LPS challenge. (A) Examination of NO levels with related kits. (B) Western blot analysis of p‐AKT and eNOS expression. HPMVEC, human pulmonary microvascular endothelial cell. **p < .01, ***p < .001.

Next, the role of P2Y12R in migration, NO, p‐AKT, and eNOS levels were investigated. As expected, migration, as well as NO, p‐AKT, and eNOS levels were decreased by LPS stimulation when compared with the control group. These impacts were significantly reversed by ticagrelor or clopidogrel treatment (Figures 3 and 4A,B). These results suggested the protective role of P2Y12R antagonists in LPS‐suppressed migration and LPS‐induced dysfunction of HPMVECs.

### 3.3 P2Y12R antagonists reduce LPS‐induced permeability of HPMVECs

**Figure 5:**
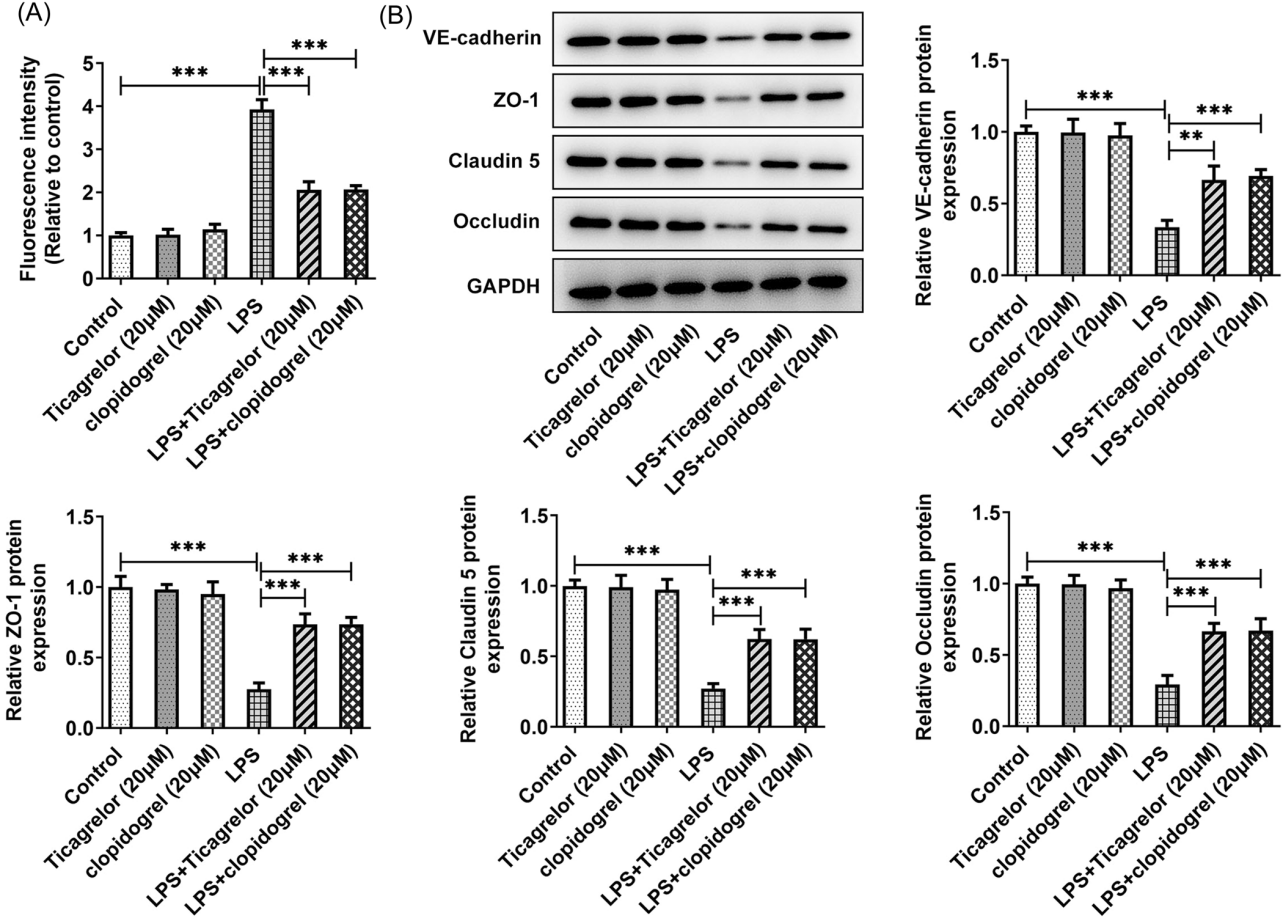
Ticagrelor or clopidogrel treatment decreases permeability of LPS‐treated HPMVECs. (A) The evaluation of cell permeability by FITC‐dextran. (B) Western blot analysis of VE‐cadherin, ZO‐1, Claudin 5, and Occludin. HPMVEC, human pulmonary microvascular endothelial cell. **p < .01, ***p < .001.

Further, whether ticagrelor or clopidogrel treatment could influence the permeability of HPMVECs challenged with LPS was investigated. The endothelial cell permeability was detected through FITC‐dextran. There were no significant changes in endothelial cell permeability in ticagrelor or clopidogrel‐treated HPMVECs. However, there was a significant decrease in endothelial cell permeability in HPMVECs cotreated by LPS and ticagrelor or clopidogrel (Figure 5A). Additionally, ticagrelor or clopidogrel treatment significantly upregulated the expression of VE‐cadherin, ZO‐1, Claudin 5, and Occludin compared with the LPS group (Figure 5B).

## 4 DISCUSSION

LPS‐induced inflammation, decreased migration, and endothelial dysfunction were significantly affected by P2Y12R antagonists, suggesting that P2Y12R participated in lung injury. Decreased levels of NF‐κB p65 in nuclei, and marked reduction in the phosphorylation levels of IκB‐α were observed, suggesting that P2Y12R inhibition had an inhibitory effect on NF‐κB/IκB‐α signaling pathway. NF‐κB inhibition is likely to be related to increased levels of NO and phosphorylation levels of eNOS by ticagrelor or clopidogrel treatment. The increased expression of eNOS and increased content of NO can downregulate NF‐κB expression.^14^ NO production dependent on eNOS is capable of mediating NF‐κB activation, inflammation, lung microvascular permeability, and edema formation induced by LPS.^15^ Thus, the increase in NO production by ticagrelor or clopidogrel treatment could result in the inhibition of NF‐κB. Additionally, ticagrelor or clopidogrel treatment increased phosphorylation levels of AKT in HPMVECs after LPS challenge.

AKT‐related signaling pathway plays an important regulatory role in cell survival, proliferation, differentiation, migration, and other cell functions, and is an important compensation mechanism for the body to cope with various harmful stimuli.^16^, ^17^ eNOS is an important downstream target of AKT and plays an important role in regulating vascular growth and endothelial function.^18^ AKT/eNOS signaling pathway is engaged in proliferation, migration, and inflammation in LPS‐induced cell dysfunction.^19^, ^20^ AKT/eNOS signaling pathway is involved in mediating the protective effect of pulmonary vascular endothelial barrier in mice with ARDS.^19^ It was also observed that ticagrelor or clopidogrel treatment suppressed the inflammation and promoted the migration of LPS‐challenged HPMVECs. This result pointed out that P2Y12R might regulate AKT/eNOS/NO signaling pathway to involve in LPS‐induced cell injury.

It has been reported that the inhibition of VE‐cadherin transcription, which exists in vascular endothelium, is the basis of inflammatory lung injury induced by sepsis.^21^, ^22^ In sepsis‐induced lung injury, there is abnormal decrease in the expression of ZO‐1, Claudin 5, and Occludin, which are involved in lung vascular permeability.^23^, ^24^ Increased endothelial permeability and expression of VE‐cadherin, ZO‐1, Claudin 5, and Occludin after ticagrelor or clopidogrel treatment was observed in HPMVECs with LPS induction, suggesting that P2Y12R affected endothelial barrier dysfunction after LPS challenge.

In conclusion, the data showed that P2Y12R antagonists protected HPMVECs against LPS‐induced injury by decreasing inflammation and promoting migration, as well as improving endothelial cell function and permeability, demonstrating that P2Y12R played a vital role in ALI, and this finding might provide a new sight for the treatment of ALI. Nonetheless, there are also some limitations here. It was demonstrated that P2Y12R inhibitors could improve ALI from a macroscopic perspective in this study. Target genes of P2Y12R inhibitors need to be identified and whether aberrant expression of target genes will affect ALI also needs to be explored.

## CONFLICT OF INTEREST

The author declares no conflict of interest.

## Data Availability

The data sets used and/or analyzed during the current study are available from the corresponding author upon reasonable request.
